# Multi-Class Skin Lesion Classification Using a Lightweight Dynamic Kernel Deep-Learning-Based Convolutional Neural Network

**DOI:** 10.3390/diagnostics12092048

**Published:** 2022-08-24

**Authors:** Theyazn H. H. Aldhyani, Amit Verma, Mosleh Hmoud Al-Adhaileh, Deepika Koundal

**Affiliations:** 1 Applied College in Abqaiq, King Faisal University, P.O. Box 400, Al-Ahsa 31982, Saudi Arabia; 2 School of Computer Science, University of Petroleum & Energy Studies, Dehradun 248007, India; 3 Deanship of E-Learning and Distance Education, King Faisal University, P.O. Box 4000, Al-Ahsa 31982, Saudi Arabia

**Keywords:** deep learning, skin diseases, biomedical image, artificial intelligence

## Abstract

Skin is the primary protective layer of the internal organs of the body. Nowadays, due to increasing pollution and multiple other factors, various types of skin diseases are growing globally. With variable shapes and multiple types, the classification of skin lesions is a challenging task. Motivated by this spreading deformity in society, a lightweight and efficient model is proposed for the highly accurate classification of skin lesions. Dynamic-sized kernels are used in layers to obtain the best results, resulting in very few trainable parameters. Further, both ReLU and leakyReLU activation functions are purposefully used in the proposed model. The model accurately classified all of the classes of the HAM10000 dataset. The model achieved an overall accuracy of 97.85%, which is much better than multiple state-of-the-art heavy models. Further, our work is compared with some popular state-of-the-art and recent existing models.

## 1. Introduction

The largest organ in the human body is the skin, which is composed of many layers (the epidermis, the dermis, the subcutaneous tissues, the blood vessels, the lymphatic vessels, the nerves, and the muscles). The ability of the skin to act as a barrier can be strengthened by employing fluids to stop the breakdown of lipids in the epidermis. Diseases of the skin can be brought on by a fungus that grows on the skin, bacteria that are not visible to the naked eye, allergic responses, bacteria that change the texture of the skin, or pigmentation [1]. Chronic skin conditions can develop into cancerous tissues on rare occasions. Skin disorders must be treated as soon as they appear to keep them from spreading and progressing [2]. Imaging-based treatments for determining the consequences of various skin diseases are in high demand. It may take months before a patient is diagnosed with the signs of several skin illnesses, making treatment difficult. There has been a lack of generalization in previous dermatological computer-aided categorization works due to a lack of data and a concentration on routine tasks such as dermoscopy, which refers to the examination of the skin utilizing a microscope of the skin surface. Computer-aided diagnosis can be used to diagnose skin illnesses and provide treatment based on the symptoms of patients [3]. Skin illnesses can be accurately identified using supervisory procedures that reduce the cost of diagnosis. The progression of sick growth is monitored using a grey-level co-occurrence matrix. For more effective treatment and lower pharmaceutical costs, it is critical that a diagnosis be accurate.

There is a big disparity between those who have skin illnesses and those who have the training to treat them. Dermatologists, equipment, drugs, and researchers are among the resources available. Those living in rural areas suffer the most from a lack of resources, according to the World Health Organization. Automated expert systems for the classification of early skin lesions are necessary because of the massive imbalance between the skin patients and the expertise. In resource-constrained locations, these categorization algorithms can aid in the early detection of skin lesions [4,5]. Computer vision algorithms have been offered in the literature as comprehensive research solutions for early skin lesion diagnosis and the aforementioned complexity [6]. DTs, SVMs, and ANNs are just a few examples of the many different approaches available for classifying data [7,8]. In Reference [9], a comprehensive evaluation of various strategies is provided. As a result, many machine learning approaches rely on photos with low noise and high contrast that cannot be used with skin cancer data. Color, texture, and structural traits play a role in skin classification. As skin lesions have a significant degree of inter-class homogeneity and intra-class heterogeneity, the classification may lead to incorrect findings with weak feature sets [10]. Since skin cancer data are not normally distributed, the usual methodologies cannot use them because they are parametric. These approaches are ineffective since each lesion has a unique pattern. Using deep learning approaches in skin classification, dermatologists can accurately diagnose lesions. Deep learning’s role in medical applications has been explored in depth in some studies [11,12].

Basal cell carcinoma, squamous cell carcinoma, and melanocyte carcinoma are the most common subtypes of skin cancer [13]. The most prevalent kind of cancer, known as basal cell carcinoma, is characterized by sluggish progression and does not metastasize to other areas of the body. Because it often comes back, getting rid of it thoroughly from the body is essential. Squamous cell carcinoma is a different type of skin cancer that can spread to other places of the body and goes deeper into the skin than basal cell carcinoma. When the skin is exposed to sunlight, melanocytes, the cells that make the skin dark or tan, create melanin. Cancerous moles, which are also known as melanoma cancer, arise when the melanin within these cells accumulates in the body. Melanocyte-based malignancies, on the other hand, are classified as malignant and can be life-threatening due to their ability to inflict minor damage to surrounding tissues. The ISIC Skin Imaging Collaboration is one of the datasets that has been utilized rather frequently for the purpose of this study [14]. According to the data provided by the ISIC 2016–2020, lesions may be broken down into the following four categories: The most frequent types of skin lesions are known as nevus (NV), seborrheic keratosis (SK), benign (BEN), and malignant (MEL). The trunk, arms, and legs can all display varying hues of pink, brown, and tan that indicate NV cancer. The next kind is the SK, which, when it is not malignant, can have the appearance of a waxy brown, black, or tan. BEN is a sort of lesion that is not malignant and does not penetrate the tissues that are nearby, nor does it spread to other parts of the body. A lesion is said to be BEN if it possesses both NV and SK components. MEL is a massive brown mole with dark speckles that can bleed or change color over time. This is the final and most important point. It is a cancer that is quite aggressive and quickly spreads throughout the body. There are several subtypes of MEL, including acute, nodular, and superficial. The purpose of this research is to differentiate between MEL and BEN cancers, which is the study’s primary objective. Our major contributions are as follows: (i) An improved convolutional neural network is proposed by using variable size kernels and activation function in the network. Moreover, fewer numbers of kernels are used in the first three layers of the network as compared to the last two layers, which results in efficient utilization of kernels. (ii) The ReLU activation function is used in the first three layers of the network, whereas leakyReLU is used in the last two layers of the convolutional neural network to improve the performance of the skin lesion classification. (iii) Class-wise balancing of data has been performed to unbiased the training. (iv) The model has achieved high accuracy with fewer parameters and in less computational time as compared to other state-of-the-art models and existing works. 

## 2. Background of Study

There is a huge number of deaths each year due to skin cancer, which is prevalent all over the world [15]. To preserve lives, it is critical to perform early identification of this aggressive disease. The ABCDE [16] criteria are followed by several histopathology tests by clinical professionals. Preprocessing, feature extraction, segmentation, and classification are some of the standard processes that can be automated using artificial intelligence-based algorithms. Handcrafted feature sets, which lack generalizability for dermoscopic skin pictures, were heavily relied upon in several classification algorithms [17,18]. Because of their similarities in color, shape, and size, lesions are highly linked, resulting in inadequate feature information [19,20].

In order to extract features, the ABCD scoring method was applied to the data. Lesion classification was completed by employing a combination of existing approaches. The thickness of the lesion was used to classify melanoma in [21]. First, lesions were classified as thin or thick, and second, they were classed as thin, medium, and thick. The logistic regression and artificial neural networks were proposed for classification purposes. To increase the number of lesions, a median filter was applied in a distinct manner to each of the RGB channels [22]. In order to segregate these lesions, a deformable model was utilized. The Chan–Vese model was used as the foundation for a segmentation approach that was developed in [23].

A support vector machine was used to classify these features support vector machine (SVM). The paraconsistent logic (PL) method was used by the authors to classify melanoma (MEL) and basal cell carcinoma (BCC) [24]. They were able to determine the strength of the evidence, the pattern of formation, and the diagnostic contrast. BCC and MEL were distinguished using spectra with values of 30, 96, and 19, respectively. In [25], the binary mask of ROIs was extracted using a Delaunay Triangulation. By removing the granular layer boundary, the authors of [26] were able to identify only two lesions in the histological pictures. Alam et al. [27] presented an SVM to automate the detection of eczema. This was accomplished by segmenting the acquired image, choosing features based on texture-based information for more accurate predictions, and ultimately utilizing the support vector machine (SVM) for evaluating the advancement of eczema as reported by I. Immagulate [28]. When dealing with noisy image data, it is inappropriate to apply the support vector machine modeling technique [29]. When working with an SVM, it is essential to locate parameter values that are feature-based. If there are more parameters in each feature vector than there are data samples that were utilized for training, then its performance will be subpar.

Artificial neural networks and convolutional neural networks (CNN) are the methods that are employed most frequently for artificial neural networks to detect and diagnose abnormalities in radiological imaging data [30,31]. The CNN method of diagnosing skin diseases has produced good results [32]. This makes working with images taken on a smartphone or digital camera difficult because CNN models are not scaled or rotation invariant. Both neural network approaches require enormous amounts of training data to achieve the model’s high performance, which in turn necessitates a substantial amount of computational effort [33]. The models based on neural networks are more abstract, and we are unable to modify them to suit our own requirements because of this. Additionally, the number of trainable parameters in ANN skyrockets as picture resolution improves, which necessitates massive training efforts in order to achieve accurate results. The gradient shrinks and explodes, which causes problems for the ANN model. In CNN’s findings, the object’s magnitude and size are not correctly interpreted [34,35].

J. Zhang et al. [35] proposed CNN for skin classification. A deep convolutional neural network (DCNN) was used to investigate the network’s inherent ability to pay attention to itself. With the use of attention maps at lower layers, each ARL block develops residual learning mechanisms that help it better categorize input data. On the basis of the ISIC 2017-19 datasets, Iqbal et al. [36] developed a DCNN model for classifying multi-class skin lesions. In the beginning, the model transmits feature information from the top to the bottom of the network; their model employs 68 convolutional layers, which are made up of interconnected blocks. In addition, a similar approach was used by Jinnai and colleagues [37]. They classify melanoma using 5846 clinical photos rather than dermoscopy using the FRCNN algorithm. To prepare the training dataset, they manually drew borders around lesion locations. Ten board-certified dermatologists and ten dermatology trainees were outperformed by the FRCNN, which had a better level of accuracy. Barata et al. [38] offered an inquiry into boosting the performance of the ensemble CNN model in terms of accuracy by developing the proposed model. The fusion of data generated by four separate classification layers was utilized to create an ensemble model for three class classifications from GoogleNet, AlexNet, VGG, and ResNet Classification accuracy can be improved by taking into account the patient’s metadata as proposed by Jordan Yap et al. [39]. Dermoscopic and macroscopic pictures were both sent into the ResNet50 network and then were utilized to classify them together. Multimodel classification outperformed the simple macroscopy-based model with an AUC of 0.866. The ISIC 2019 dataset was used by Gessert et al. [40] to develop an ensemble model that incorporated EfficientNet, SENet, and ResNeXt WSL. They used a cropping approach to deal with photos having different resolutions from different models. In addition, a technique of loss balancing was created to deal with datasets that were unbalanced. On the HAM10000 dataset, Srinivasu et al. [41] classified lesions by employing a deep convolutional neural network (DCNN) equipped with MobileNetV2 and long short-term memory (LSTM). MobileNetV2 was a CNN model that offered several advantages over existing CNN models, including a cheaper computational cost, a smaller network size, and compatibility with mobile devices. In the LSTM network, the features of MobileNetV2 were each given a timestamp as they were stored. When MobileNetV2 was combined with LSTM, there was an improvement in accuracy of up to 85.34 percent. 

A straightforward and efficient method for improving images is known as histogram equalization. Because the equalizing method has the potential to dramatically alter the luminance of a picture in certain circumstances, it has never before been used in a video system; this is the reason why the technology has never been used. In this research, a novel histogram equalization method known as equal area dualistic sub-image histogram equalization is proposed [42].

Huang et al. [43] proposed deep learning techniques to create a lightweight model for classifying skin cancer that might be used to improve medical care. In this study, they looked at the clinical images and medical records of patients who had received a histological diagnosis of basal cell carcinoma, squamous cell carcinoma, melanoma, seborrheic keratosis, or melanocytic nevus in the Department of Dermatology at Kaohsiung Chang Gung Memorial Hospital between the years 2006 and 2017. In order to develop a skin cancer classification model, they used deep learning models to differentiate between malignant and benign skin tumors in the KCGMH and HAM10000 datasets. This was accomplished by binary classification and multi-class classification. In the KCGMH dataset, the deep learning model achieved an accuracy of 89.5% for binary classifications (benign vs. malignant), whereas in the HAM10000 dataset, the accuracy of the deep learning model was 85.8%.

Thurnhofer-Hemsi et al. [44] introduced a deep learning model, namely MobileNet V2 and long short-term memory-based deep learning, to identify skin cancer. Experiments were conducted on the HAM10000 dataset, a sizable collection of dermatoscopic images, with the aid of data augmentation techniques to boost results. The investigation’s findings indicate that the DenseNet201 network is well suited for the undertaking at hand, as it achieved high classification accuracies and F-measures while simultaneously reducing the number of false negatives [45]. 

Ioannis Kousis et al. [46] presented a convolutional neural network (CNN) for detecting skin cancer; using the HAM10000 dataset, they trained and evaluated 11 different CNN architectures for identifying seven distinct types of skin lesions. In order to combat the imbalance issue and the great similarity between images of some skin lesions, they employed data augmentation (during training), transfer learning, and fine-tuning. According to their results, the DenseNet169 transfer model outperformed the other 10 CNN architecture variants. 

## 3. Materials and Methods

### 3.1. Dataset

**Figure 1:**
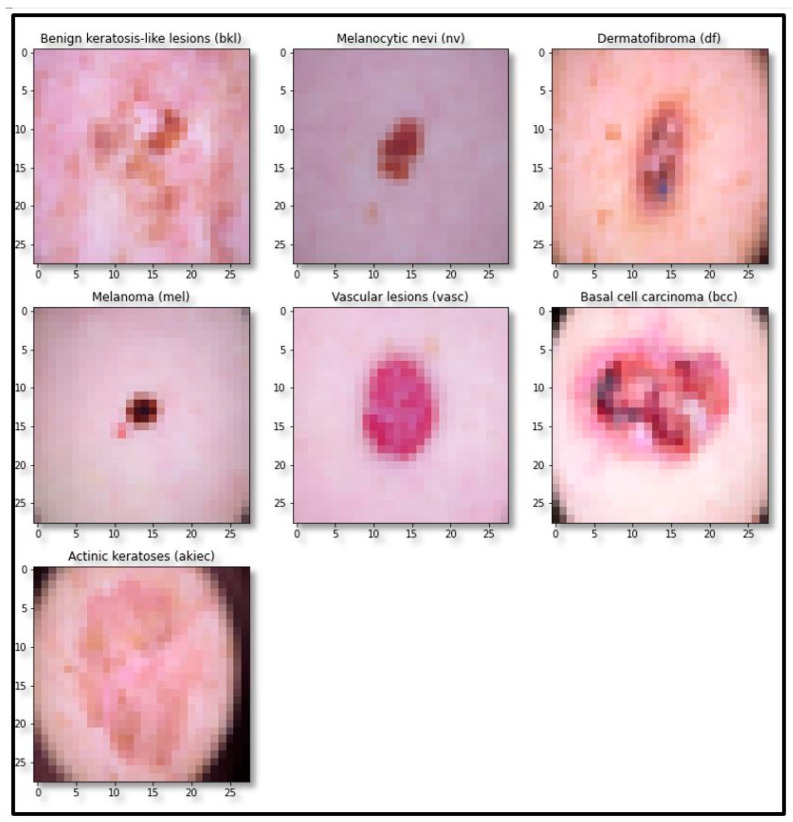
Sample images of each class of the dataset.

The standard skin lesion dataset HAM10000 was used for experimentation. It contains 10,015 skin lesion images of divergent populations distributed in seven major classes, as shown in Table 1. The sample image of each class is shown in Figure 1. 

### 3.2. Data Balancing and Augmentation

**Figure 2:**
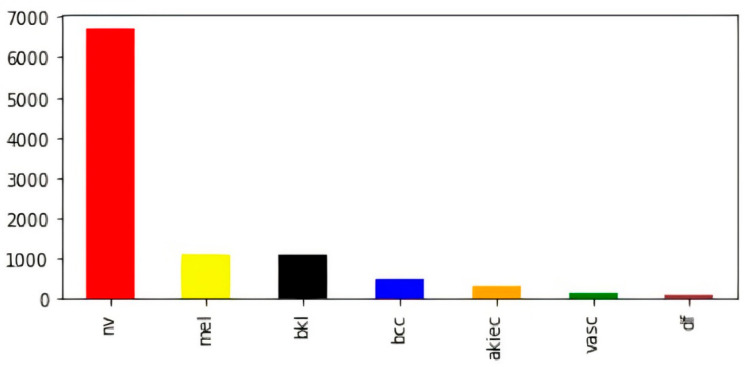
Class-wise count of images in original dataset (before balancing).

To overcome variable-sized images in the dataset, all images were resized to (28, 28, 3). In addition to this, we found a significant disparity in the numbers of images contained inside the various classes. For example, the melanocytic nevi (mel) class has a large number of samples as compared to the rest of the classes. Further, vascular lesions (vasc) and dermatofibroma (df) classes have fewer samples. The bar visualization of the class-wise sample distribution of the original dataset is shown in Figure 2; however, to efficiently train a deep-learning-based model, we require a reasonable amount of balanced data. Further, the imbalanced data may cause the model training to remain biased towards some particular classes with a comparatively large number of samples; therefore, to avoid biased training of the model, data balancing is performed by artificially generating the required samples [47,48,49]. 

**Figure 3:**
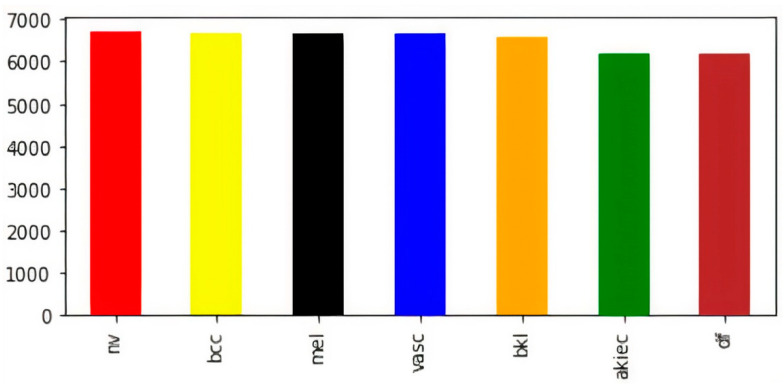
Class-wise count of images after balancing.

The visual representation of class-wise images in the balanced dataset is shown in Figure 3. Now, the dataset has an almost equal number of images in every class of skin lesions, which ensures the unbiased training of the model. Now, for the sake of training, testing, and validation, we perform a random split of the data, as shown in Table 2.

After splitting the dataset, augmentation is performed to improve the robustness of the trained model so that the model can perform adequately for unseen samples; therefore, training samples are horizontally and vertically flipped, randomly rotated (−10 to +10), horizontally and vertically shifted (0.2), zoomed-in and zoomed-out (20%), and empty areas in the samples are filled by nearest pixels; whereas testing data samples are augmented to estimate the performance of the presented model over actual data. We perform random rotation for smaller angles as we are already performing horizontal and vertical flips.

### 3.3. Architecture Overview

**Figure 4:**
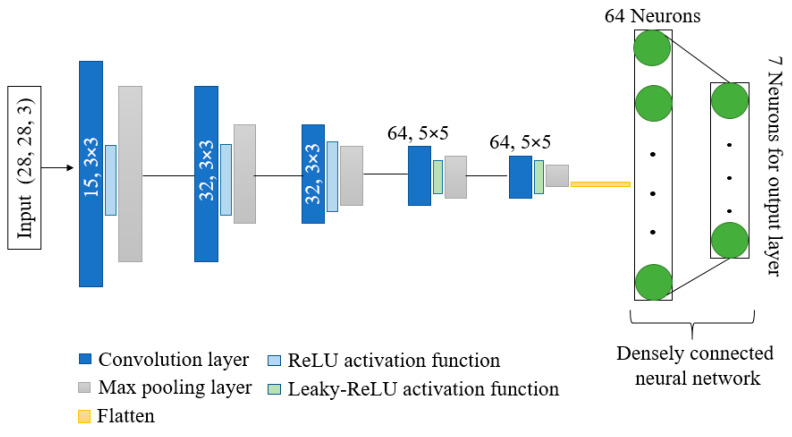
Architecture of proposed DKCNN model.

Figure 4 depicts the overall design of the model that is being suggested. The model has shown better performance with fewer parameters and computation time as compared to other state-of-the-art models. Kernels and activation functions play a vital role in the training of any convolution-based model. Performance and resource utilization of the model depends on the size and number of kernel and activation functions used in the network. Motivated by the astounding abilities of kernels and activation functions, we have proposed a network with dynamic-sized kernels and two different activation functions in the network. The network has five layers; each layer comprises convolution operation, activation function, and pooling operation. The input of shape 28 × 28 × 3 is given to the first layer of the network, and the first layer has 15 kernels and each of size 3 × 3. Fewer kernels are used in the first layer. The starting layer’s kernels are used to identify some basic features only; therefore, using fewer kernels reduces the number of parameters, computation times, and can easily identify basic features. During convolution operation, the padding remains the same so that the size of the feature maps remain the same as the input image. These feature maps are passed through the ReLU activation function to reduce the linearity. Further, max pooling is applied on the feature maps with a pool size of 2 × 2. In the pooling layer also, padding is the same to avoid input dimension problems during max pooling operation. In the starting layers, the feature maps contain fewer complex features; therefore, we used the ReLU activation function to reduce linearity. Further, in the starting layers, we have a slight chance of information loss due to the activation function. In the second layer, we increase the number of kernels from 15 to 32 to extract more complex features as compared to layer 1, and used the ReLU activation function and max pooling with a pool size of 2 × 2.

The configuration of layer 3 is the same as layer 2. In layers 4 and 5, we further increased the number of kernels from 32 to 64 with a size of 5 × 5. Further, the leakyReLU activation function is used instead of the ReLU activation function. In the last two layers, to obtain more complex features, we have increased the number of parameters by increasing the kernel size and number of kernels. 

LeakyReLU is preferred in the last two layers due to its ability to maintain non-linearity in the feature maps and to reduce the chances of information loss of salient features in the feature maps. Moreover, the output of the last layer is flattened and provided as input to the densely connected neural network. The input layer of the dense network has 64 neurons, and ReLU is used as an activation function. In contrast, the output layer has 7 neurons, equal to the number of classes in the dataset with a SoftMax activation function.

## 4. Result and Discussion

This section provides comprehensive information about the dataset, the process of data balancing and augmentation, experimental setup, evaluation metrics and performance analysis, and comparative studies with various state-of-the-art methods.

### 4.1. Evaluation Metrics and Performance Analysis

Precision, recall, f1-score, and accuracy are calculated to evaluate the performance of the trained model with testing samples. Testing samples are skin lesion images without augmentation. The objective here is to know the performance of the model for unseen samples. Mathematical formulas for calculating these metrics are shown in Equations (1)–(4).

(1) $$Accuracy\;\left(ACC\right)=\frac{TP}{TP+TN+FP+FN}$$

(2) $$Precesion\;\left(PRE\right)=\frac{TP}{TP+FP}$$

(3) $$Recall\;\left(REC\right)=\frac{TP}{TP+FN}$$

(4) $$f1-score=\frac{2*TP}{2*TP+FP+FN}$$

**Figure 5:**
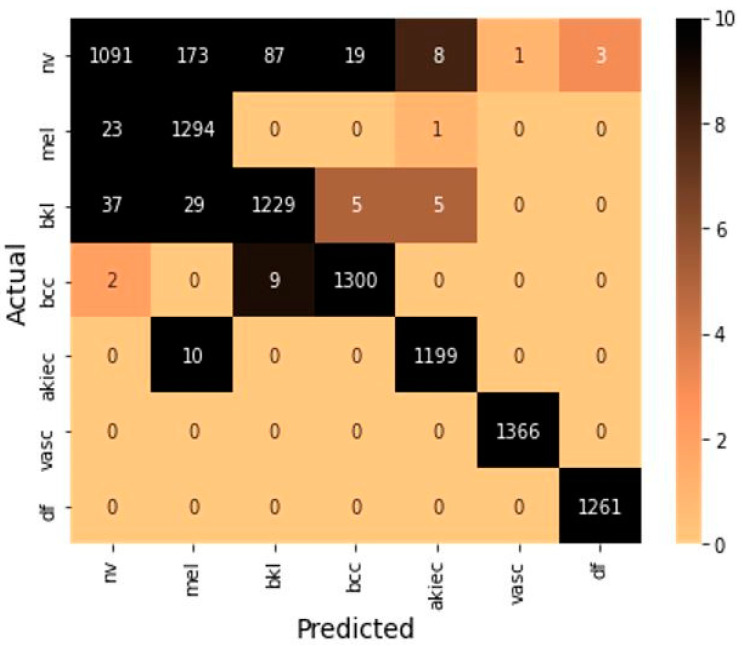
Confusion matrix on testing samples.

These formulas include true positive (*TP*) and true negative (*TN*), which represent a number of positive and negative samples identify correctly. Further, false positive (*FP*) and false negative (*FN*) indicate the number of positive and negative samples identified incorrectly. The confusion matrix is shown in Figure 5 to summarize the class-wise prediction information of the model. Providing the number of *TP*, *TN*, *FP*, and *FN* predicted by the model also highlighted the non-zero numbers in ascending order with increasing color density to improve the readability of the confusion matrix.

Model is trained using Adam optimizer for 20 epochs with a batch size of 64. With efficient utilization of number and kernel size, very few trainable parameters are used to train the model with an initial learning rate of 0.001. The model is broadly summarized in Table 3. Activation function ReLU is used in some starting layers of the model, whereas leakyReLU is used in the last two layers before flattening.

**Figure 6:**
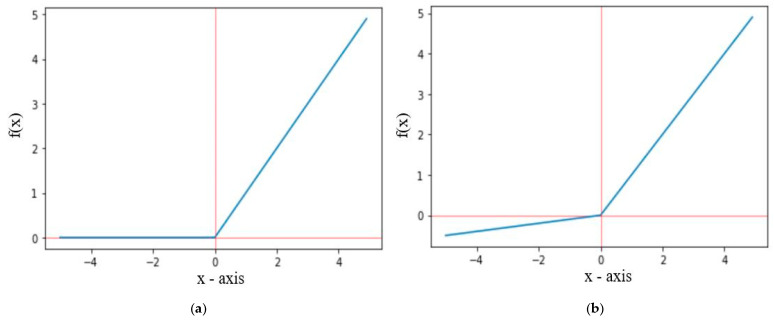
Graph representation of (**a**) ReLU and (**b**) LeakyReLU.

The ReLU activation function only propagates positive values received from previous layers to output or hidden layer, whereas the leakyReLU activation function allows some small negative values. The graphical representation of ReLU and leakyReLU are shown in Figure 6, where *f*(*x*) for ReLU and leakyReLU activation functions are defined in Equations (5) and (6), respectively. For leakyReLU α = 0.01, which allows a small part of negative values instead of 0.

(5) $$f\left(x\right)=\{\begin{array}{c} x\;\;\;for\;\;\;x\geq 0\\ \;\;0\;\;\;for\;\;\;x<0 \end{array}$$

(6) $$f\left(x\right)=\{\begin{array}{c} x\;\;\;for\;\;\;x\geq 0\\ \;\;\alpha x\;\;\;for\;\;\;x<0 \end{array}$$

**Figure 7:**
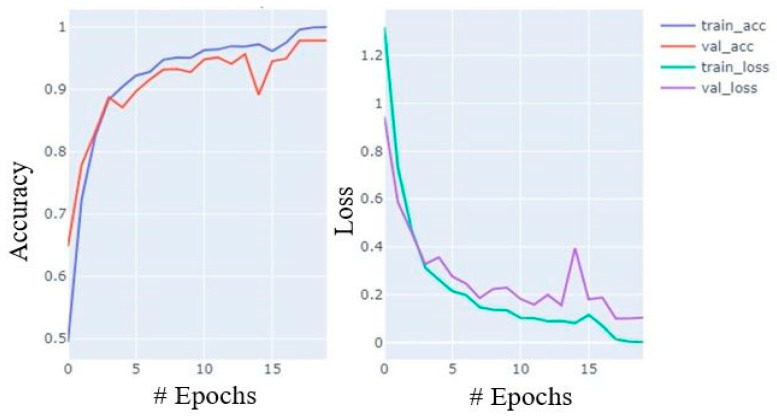
Accuracy and loss plot during training process with training and validation data.

During training, the accuracy of the model increases with celerity until the 7th epoch; thereafter, it increases at a moderate pace until the 15th and the growth slows from the 16th to the 20th. Similarly, loss also decreases in almost similar paces. The accuracy and loss curves for training and validation data are shown in Figure 7.

The model has shown remarkable performance over unseen tenting samples. The class-wise accuracy (ACC), precision (PRE), recall (REC), and f1-score of the model are shown in Table 4. The model obtained 97.858% as the overall accuracy. Further, the performance of the model was evaluated using the k-fold cross-validation approach. The dataset is divided into k = 10 equal parts (folds), the model is trained with k-1 folds (training set), and the remaining fold is used as a test set. This process continued for k times and each fold was used once as a test set. The average accuracy, precision, recall, and f1-score calculated with each fold of data using k-fold cross validation methods are shown in Table 5. 

### 4.2. Experimental Setup

The proposed model was implemented using python 3.8.8 with Keras API of the TensorFlow 2.7.0 library. The model was trained using a Jupyter notebook with a CPU for computation. Further, the model was trained separately on a Kaggle notebook using a GPU. With the efficient utilization of a number and size of kernels, the proposed model was trained in much less computation time and with only 172,363 parameters. 

The training times of the model with CPU and GPU are 375.17 and 82.48 s, respectively. We have used an Adam optimizer with a batch size of 64, 20 epochs, and an initial learning rate of 0.001, which was reduced to 0.0001 after the 11th epoch.

### 4.3. Comparison with Some State-of-the-Art Methods and Other Existing Models

The performance of the model in terms of the number of parameters, precision, recall, and f1-score are compared with some existing models over a similar dataset, which is shown in Table 6. It has been found that the proposed model has shown outstanding performance with very few trainable parameters and computation time.

## 5. Conclusions and Future Scope

Nowadays, skin disease is a global challenge affecting a major proportion of the world’s population. Multiple classes of skin disease may extend from minor to major types of involved danger. Skin cancer is rapidly increasing and becoming a significant health concern. Correct and timely treatment plays a pivotal role in reducing this life threat; therefore, an accurate and fully automatic classification system is a major requirement of the medical field; however, there are multiple machine learning and deep-learning-based methods that have been proposed to solve this purpose. As per the literature review, we find opportunities to propose a better lightweight model with high accuracy. As a result, we proposed a CNN-based model with efficient utilization of kernels and activation functions. The proposed model has shown a remarkable class-wise (seven classes) accuracy and overall accuracy of 97.85% on the test dataset with fewer parameters than is standard (172,363). The proposed model can also be used for disease classification with a dataset that has more classes. The model still has room for more accurate prediction of benign keratosis-like lesions, melanoma, and melanocytic nevi classes of skin lesions.

## Figures and Tables

**Table 1:** Distribution of skin lesion images in seven major classes and total images in the dataset.

Class	Count
Melanocytic nevi (nv)	6705
Basal cell carcinoma (bcc)	1113
Melanoma (mel)	1099
Vascular lesions (vasc)	514
Benign keratosis-like lesions (bkl)	327
Actinic keratoses (akiec)	142
Dermatofibroma (df)	115
Total	10,015

**Table 2:** Distribution of skin lesions in train and testing data.

Classes	nv	mel	bkl	bcc	akiec	vasc	df	Total
# Training samples	5323	5360	5289	5371	5004	5308	4949	36,604
# Testing samples	1382	1318	1305	1311	1209	1366	1261	9152
# Validation samples	1338	1391	1303	1314	1271	1295	1239	9151

# represents”Number of”.

**Table 3:** Broad summary of the model.

Optimizer	Batch Size	# Epochs	Activation	Optimizer	Batch Size
Adam	64	20	ReLU & LeakyReLU	0.001	172,362

# represents”Number of”.

**Table 4:** Class-wise accuracy, precision, recall, and f1-score obtained by the model.

Label	ACC	PRE	REC	F1-Score
nv	0.87	1.00	0.87	0.93
mel	0.98	0.94	1.00	0.97
bkl	0.99	0.94	0.99	0.97
bcc	1.00	0.99	1.00	0.99
akiec	1.00	1.00	1.00	1.00
vasc	1.00	1.00	1.00	1.00
df	1.00	1.00	1.00	1.00
**Average**	0.978	0.981	0.98	0.98

**Table 5:** Accuracy, precision, recall, and f1-score with each fold of data calculated using k-fold cross-validation method.

Folds	1	2	3	4	5	6	7	8	9	10
**ACC**	0.94	0.91	0.94	0.97	0.98	0.99	1.00	0.98	1.00	1.00
**PRE**	0.95	0.97	0.94	0.98	0.98	0.98	1.00	0.96	0.98	0.98
**RECALL**	0.96	0.97	0.95	0.98	0.98	0.98	1.00	0.97	0.98	0.98
**F1-score**	0.95	0.97	0.94	0.98	0.98	0.99	1.00	0.96	0.98	0.98

**Table 6:** Comparison of proposed work with some standard models and other existing models.

Ref.	Model	Data Set	ACC %
Ref. [42]	Histogram equalization	HAM10000	85.80
Ref. [43]	Deep leaning	HAM10000	89.5
Ref. [44]	DenseNet201 network	HAM10000	92.83
Ref. [45]	MobileNet + LSTM	HAM10000	85.34
Ref. [46]	DenseNet169	HAM10000	91.10
	**Proposed Work**	**HAM10000**	**97.85**

## Data Availability

The data presented in this study are available here: https://www.kaggle.com/kmader/skin-cancer-mnist-ham10000 (accessed on 17 May 2022).
